# Timely Initiation of Complementary Feeding and Associated Factors among Mothers of Children Aged 6–24 Months in Dessie Referral Hospital, Northeast Ethiopia, 2019

**DOI:** 10.1155/2020/6756202

**Published:** 2020-10-29

**Authors:** Atsedemariam Andualem, Afework Edmealem, Belachew Tegegne, Lehulu Tilahun, Yitayish Damtie

**Affiliations:** ^1^Department of Nursing, School of Nursing and Midwifery, Wollo University, Dessie, Ethiopia; ^2^Department of Emergency and Ophthalmic Health, School of Nursing and Midwifery, Wollo University, Dessie, Ethiopia; ^3^Department of Reproductive Health, School of Public Health, Wollo University, Dessie, Ethiopia

## Abstract

**Background:**

Timely, appropriate, safe, adequate, and frequent feeding is essential during the transition period for optimal growth and development as well as vulnerability of the child. The age of initiation of complementary feeding needs to be strongly addressed. Thus, the aim of this study was to determine timely initiation of complementary feeding and associated factors among mothers of children aged 6–24 months in Dessie Referral Hospital.

**Methods:**

Institutional-based cross-sectional study was conducted among 280 mothers of children aged 6–24 months. A systematic random sampling technique was employed for selection of study participants by considering the 1st comer as a starting point and then at every 5th interval till the sample size was saturated at exit time. Data were collected using pretested and validated structured interviewer-administered questionnaire. Data were entered to Epi data version 3.1 and exported to SPSS version 20.0 software for analysis. Descriptive statistics and binary logistic regression model were used.

**Results:**

Overall response rate was 98.2%. Among 275 mothers with children aged 6–24 months, 36 (13.1%), 179 (65.1%), and 60 (21.8%) mothers started giving complementary feeding for their children early (before six months), timely (at six months), and late (after six months), respectively. Mothers' educational status of grade 9–12 and college and above [AOR = 3.03; 95% CI (1.13–8.14), and AOR = 3.74; 95% CI (1.19–11.70), respectively], getting counsel [AOR = 2.83; 95% CI (1.54–5.21)], and poor knowledge [AOR = 0.37; 95% CI (0.19–0.72)] were found to be independent predictors.

**Conclusions:**

Prevalence of timely initiation of complementary feeding was high as compared to the national prevalence. Mothers' educational status, getting counsel about complementary feeding, and knowledge were factors associated with timely initiation of complementary feeding. Therefore, awareness creation, counseling, and health education should be done on society by concerned bodies to improve timely introduction of complementary feeding level more than this result.

## 1. Introduction

Complementary feeding is the process of starting foods and liquids along with breast milk when breast milk is no longer sufficient to meet nutritional requirements of children [1]. Appropriate, safe, adequately nourished, and frequent feeding is essential during the transition period from child's birth to 2 years of age since this period is a critical window for optimal growth and development as well as vulnerability of the child [2]. The World Health Organization (WHO) recommends that complementary feeding should be commenced at the age of 6 months, and the frequency of nonmilk feeding should gradually increase until 24 months. But most mothers do not start complementary feeding at appropriate time [3–6]. Children are at increased risk of malnutrition starting from six months because breast milk alone is not sufficient to give the required nutrient contents [7].

Optimal complementary feeding is associated with some concerns like what food should be given to the child and how and when it should be given. The age of initiation of complementary feeding needs to be strongly addressed due to the risk of food intolerance, stunning, and malnutrition that do not confer any growth advantage [8, 9]. A complementary feeding practice is mostly inappropriate, which seems to be strongly associated with lack of proper knowledge among mothers regarding complementary feeding initiation time. Emphasis should be given to educate mothers about breastfeeding and initiation of complementary feeding practices during immunization [10].

Evidences show that inappropriate timing of complementary feeding practices remain as major public health problem from global to national level even if it is declared that it should be initiated at 6 months' of age. This inappropriate timing of complementary feeding could result in child illness and suboptimal growth and development, and also it causes 45% child death [11, 12]. Out of 715 mothers with children, 5% of them introduced solid food for their children before 4 months of age, 14% were not started at 5.5 months of age, while 81% were introduced in between 4 and 5.5 months age [13].

Many factors can affect timely initiation of complementary feeding practice such as mother's postnatal checkup, urban residence, family size, occupation of mother, having antenatal care follow-up, believed time of initiation of complementary feeding, place of delivery, and family monthly income [14–17]. Failure to give a great attention to timely initiation of complementary feeding puts the children at risk for malnutrition. Malnutrition has been responsible, directly or indirectly 10.9 million deaths annually among children under five due to lack of timely initiation of complementary feeding [18, 19]. Despite its serious socioeconomic and health impact on children and community, it is not well studied in Ethiopia specifically in the study area. Therefore, this study was aimed to assess timely initiation of complementary feeding and associated factors among mothers of children aged 6–24 months in Dessie Referral Hospital.

## 2. Methods and Materials

### 2.1. Study Area

Dessie Referral Hospital is the only referral hospital in Wollo Province serving for about 7 million people including the neighboring regions. The hospital was established around 1840s. It covers 200 hectares of land and has 400 beds. Dessie is a major city administration in South Wollo Zone located 401 km away from Addis Ababa, the capital city of Ethiopia and 480 km from Bahir Dar, the capital city of Amhara regional state.

### 2.2. Study Design, Period, and Population

Institutional-based cross-sectional study was conducted from March 17 to April 17, 2019, among mothers of children aged 6–24 months. All eligible mothers with children aged 6–24 months in the study areas were included in the study where as those who were unable to communicate and critically ill at the time of data collection were excluded from the study.

### 2.3. Sample Size Determination and Sampling Techniques

The sample size was determined through StatCalc function of Epi Info version 7 software for each objective with the assumptions of 95% confidence interval, 5% marginal error, 80% power, unexposed to exposed ratio, percent outcome in unexposed group, and adjusted odds ratio of each major factors from the previous study [17], and the maximum calculated sample size was taken. Therefore, the maximum sample size from the Epi Info version 7 StatCalc software calculations was 280 mothers of children aged 6–24 months including nonresponse rate.

There were 1300 registered children with age of 6 to 24 months attending in Dessie Referral Hospital. The medical record numbers (MRN) of the children were used as a sampling frame. The sampling technique was systematic random sampling using the formula N ÷ n to calculate the *K* value: 1300 ÷ 280 = 5. So, mothers of children aged 6–24 months were selected by considering the 1st comers as a starting point and then at every 5^th^ interval till the sample size was saturated at exit time.

### 2.4. Data Collection and Quality Control

Data were collected through validated, structured, and pretested interviewer-administered questionnaire which was adapted from different reviewed literatures [4, 5, 15, 20–22] with some modifications by researchers. Five parts with sociodemography (14 questions), knowledge-related question (12 questions), obstetrics and gynecological-related question (9 questions), maternal reproductive history and health service utilization-related question (12 questions), and practice-related question (14 questions) (in total 61 items) were utilized for the study. The internal reliability with Cronbach's alpha values for knowledge and practice questions was 0.612 and 0.884, respectively. In order to determine the level of mothers' knowledge and practice regarding timely initiation of complementary feeding, we used mean as a cut point. Good knowledge and practice means those study participants who scored above or equal to the mean value of knowledge and practice questions, respectively. Eleven graduate nurses, eight as data collectors and three as supervisors, were recruited and trained for 2 days. To assure data quality, primarily, the questions were prepared in English and translated to Amharic, which is a local language and then retranslated back to English by bilingual experts to ensure its consistency. Principal investigators and supervisors made spot-checking and reviewing questionnaires completeness.

### 2.5. Data Management and Analysis

The collected data were checked visually for its completeness, and the responses were coded and entered into Epi data version 3.1 statistical package. Then, data were exported to SPSS version 20 for analysis. During the process of analysis, descriptive statistics were used to provide an overall and coherent presentation and description of the data. Variables with 95% confidence interval and *P* value less than or equal to 0.2 during the bivariate analysis were entered to multivariate logistic regression analysis to see the relative effect of confounding variables and interaction of variables. Odd ratio with 95% CI was performed on variables to determine the strength of association of variables. *P* value less than 0.05 was taken as the cut off value to be significant.

## 3. Results

Out of the total 280 sampled mothers with children aged 6–24 months in Dessie Referral Hospital, 275 of them were included in the study giving a response rate of 98.2%. The age of mothers of the study children ranged from 17 to 37 years with the mean ± SD of 27.44 ± 4.488 years in which most of the respondents fall within the ranges of 25–29 years age group (129 (47.0%)). Majority of the respondents had orthodox religion (132 (48.0%)), Amhara ethnicity (242 (88%)), and 4–6 family size (134 (47.8%)). As to the educational status of children' mother, 115 (41.9%) had educational status of grade 9 and above (Table 1).

### 3.1. Knowledge on Timely Initiation of Complementary Feeding

Out of the total 275 mothers with children 6–24 months of age, 263 (95.6%) knew the importance of complementary feeding and the suitability of fruits and vegetables as complementary food was 246 (89.5%). Most of mothers with children aged 6–24 months (214 (77.8%)) confirmed that complementary food should be introduced at 6 months even though all did not practice it properly due to different factors. Around 108 (39.3%) mothers did not know that adding oil to child's porridge is advisable, and 246 (89.5%) mothers knew fruits and vegetables are suitable complementary foods.

### 3.2. Complementary Feeding Practice

The prevalence of timely initiation of complementary feeding among mother with children aged 6–24 months in Dessie Referral Hospital was 65.1% with 95% CI (59.3%–70.5%). Out of the total mothers, 123 (44.7%) of them started breastfeeding for their children at the time of less than one hour after giving birth. Most of mothers started giving complementary food for their children at the age of six months 179 (65.1%). Most mothers (70 (25%)) fed semisolid (porridge) food for their children when they started complementary feeding.

### 3.3. Bivariable and Multivariable Analyses

Bivariable analysis showed significant associations between timely initiation of complementary feeding and age of mother, educational status of mother, wealth index, family size including the current child, growth monitoring follow-up, getting complementary feeding counsel, and knowledge on CF.

In multivariable logistic regression, only educational status of women, complementary feeding counseling, and knowledge on CF keep their significance association with timely initiation of complementary feeding. Therefore, a multivariable analysis model revealed that mothers having educational status of grade 9–12 and college and above were almost 3 and 4 times more likely to initiate complementary feeding timely than illiterate mothers [AOR = ; 3.03; 95% CI (1.13–8.14), *P*=0.028] and [AOR = 3.74; 95% CI (1.19–11.70), *P*=0.024], respectively. Similarly, those mothers who got counseling about timely initiation of complementary feeding were almost 3 times more likely to initiate complementary feeding timely than those who did not get [AOR = 2.83; 95% CI (1.54–5.21), *P*=0.001]. The likelihood of initiating complementary feeding timely by mothers with 6–24 months aged children having poor knowledge was almost 63% [AOR = 0.37; 95% CI (0.19–0.72), *P*=0.004] lower as compared to those mothers having good knowledge on CF (Table 2).

## 4. Discussion

In this study, the prevalence of timely initiation of complementary feeding among children in 6–24 months of age in Dessie Referral Hospital was 65.1% with 95% CI (59.3%–70.5%), which was in line with studies conducted in public health facilities found in Mekelle Town, Northern Ethiopia (62.8%) [4], and Lalibela (63%) [16]. This study finding was higher than the national prevalence (51%) [23], result from study in India (42.1%) [24], Nigeria (41%) [5], Nepal (57%) [6], and Sidama Zone Southern Ethiopia (42%) [7]. This difference might be due to sociodemographic character difference, wealth status difference, and activeness of the society. It was also low compared with study in Sodo Town, Southern Ethiopia (71.2%) [17]. This discrepancy might be due to difference in sample size, awareness of the society, and study time.

Educational status of mothers was significantly associated with timely initiation of complementary feeding; mothers having educational status of grade 9 and above were more likely to initiate complementary feeding timely than illiterate mothers. This might be due to an increment of awareness as educational status increases. This finding contradicted with studies in Nigeria [5] and Harar [25] that stated parental education was not associated with timely initiation of complementary feeding. The possible reason might be due to societal background difference and educational environment difference. And it also was consistent with study in India [24], Saudi Arabia [26], Sidama Zone, Southern Ethiopia [7], and Wollega [22], which revealed mothers who had education status of illiterate and primary school were less likely to initiate complementary feeding timely as compared to those mothers who had the educational status of high school and above.

Mothers who had got counseling about complementary feeding were more likely to initiate complementary feeding timely as compared with who had not got. This was similar with studies in Nepal [6], Sodo Town [27], and Bangladesh [28]. The possible reason might be due to the reality that person can be changed by their awareness if they get education and counseling.

The likelihood of initiating complementary feeding timely by mothers having poor knowledge was lower as compared to those mothers having good knowledge. These results were supported by study findings in Nepal [10], Pakistan [20], and Harar [25]. This may be because timely initiation of complementary feeding needs a critical knowledge on the adequacy, component, duration, and time at which it should be started; if mothers' knowledge is poor, it is difficult to initiate proper CF timely.

## 5. Conclusion

The present study showed that timely initiation of complementary feeding among children with 6–24 months of age in Dessie Referral Hospital was higher than the critical point of the national prevalence that is good. Mothers' educational level, counseling about complementary feeding, and knowledge on timely initiation of CF were found to be statistically significant with timely initiation of complementary feeding. So it is recommended that there should be awareness creation on society by counseling and giving health education to boom their knowledge and practice of timely initiation of complementary feeding.

## Figures and Tables

**Table 1:** Frequency and percentage distribution of sociodemographic characteristics of mother with children aged 6–24 months in Dessie Referral Hospital, Northeast Ethiopia, 2019 (*n* = 275).

Variables	Category	Frequency (*n* = 275)	Percentage (100%)
Age of mother in years	15–19	3	1
	20–24	58	21
	25–29	129	47
	≥30	85	31

Religion	Orthodox	132	48
	Muslim	125	45.4
	Protestant	9	3.3
	Others	9	3.3

Educational status of mother	Illiterate	75	27.2
	Read and write	45	16.4
	Grade 1–8	40	14.5
	Grade 9–12	59	21.5
	College and above	56	20.4

Occupation of mother	Housewife	130	47.3
	Government employed	54	19.6
	Private organization employed	10	3.6
	Merchant	54	20.4
	Daily laborer	9	3.3
	Others	16	5.8

Ethnicity	Amhara	242	88
	Oromo	14	5.1
	Tigre	11	4
	Afar	8	2.9

Marital status	Single	3	98.1
	Married	270	1.1
	Divorced	1	0.4
	Widowed	1	0.4

If married, educational status of husband	Illiterate	62	22.9
	Read and write	36	13.3
	Grade 1–8	32	12.2
	Grade 9–12	64	23.6
	College and above	76	28

Occupational status of husband	Government employed	74	27.2
	Private organization employed	30	11.1
	Merchant	52	19.2
	Daily laborer	57	21
	Farmer	53	20
	Others	4	1.5
	I do not know	11	4

Family size including the current child	1–3	132	48
	4–6	134	48.7
	7–10	9	3.3

Persons who live with mother in addition to husband and children	Mother's father/mother/sister/brother/relative	20	7.3
	Husband's father/mother/brother/sister/relatives	25	9.1
	Others	230	83.6

Wealth index	Poor	81	29.5
	Middle	120	43.6
	Rich	74	26.9

Age of the child in months	6–10	136	50.2
	11–15	90	31.9
	16–19	34	12.4
	20–24	15	5.5

Sex of the child	Male	146	53.1
	Female	129	46.9

**Table 2:** Factors associated with timely initiation of complementary feeding among mothers of children aged 6–24 months in Dessie Referral Hospital, Northeast Ethiopia, 2019 (*n* = 275).

Variables		Timely initiation of complementary feeding practice		COR (95% CI)	AOR (95% CI)
		Yes	No		
		*N* (%)	*N* (%)		
Age of mother	15–19	9 (75.0)	5 (25.0)	1.39 (0.35–5.49)	—
	20–24	25 (48.1)	27 (51.9)	0.43(0.22 − 0.86)^*∗*^	—
	25–29	74 (65.5)	39 (34.5)	0.88 (0.49–1.56)	—
	≥30	65 (68.4)	31 (31.6)	1.00	—

Educational status of women	Illiterate	37 (49.3)	38 (50.7)	1.00	1.00
	Read and write	23 (51.1)	22 (48.9)	1.07 (0.51–2.25)	1.29 (0.53–3.16)
	Grade 1–8	26 (65.0)	14 (35.0)	1.91 (0.86–4.21)	1.60 (0.60–4.24)
	Grade 9–12	43 (72.9)	16 (27.1)	2.76 (1.33 − 5.73)^*∗*^	3.03(1.13 − 8.14)^*∗∗*^
	College and above	10 (17.9)	46 (82.1)	4.72(2.08 − 10.73)^*∗*^	3.74(1.19 − 11.70)^*∗∗*^

Wealth index	Poor	24 (29.6)	57 (70.4)	0.14 (0.09 − 0.39)^*∗*^	—
	Middle	71 (59.2)	49 (40.8)	0.47 (0.26 − 0.89)^*∗*^	—
	Rich	60 (81.1)	14 (18.9)	1.00	—

Family size including the current child	1–3	71 (53.8)	61 (46.2)	1.00	—
	4–6	98 (73.1)	36 (26.9)	2.34 (1.40 − 3.91)^*∗*^	—
	7–10	6 (66.7)	3 (33.3)	1.72 (0.41–7.16)	—

Growth monitoring follow-up	Yes	5 (18.5)	22 (81.5)	2.73 (1.00 − 7.46)^*∗*^	—
	No	95 (38.3)	153 (61.7)	1.00	—

Complementary feeding counseling	Yes	122 (77.2)	36 (22.8)	3.90 (2.31 − 6.58)^*∗*^	2.83 (1.54 − 5.21)^*∗∗*^
	No	53 (46.5)	61 (53.5)	1.00	1.00

Knowledge level on CF	Poor	34 (53.1)	30 (46.9)	0.53 (0.29 − 0.91)^*∗*^	0.37 (0.19 − 0.72)^*∗∗*^
	Good	66 (31.3)	145 (68.7)	1.00	1.00

*∗*Variables having a *P* ≤ 0.2 in bivariable analysis; *∗∗*statistically significant at *P* value <0.05 in the multivariable analysis. COR: crude odds ratio; AOR, adjusted odds ratio; 1.00 = reference category.

## Data Availability

The datasets of this study are available from the corresponding author on reasonable request.

## Abbreviations

AOR: Adjusted odds ratio
CF: Complementary feeding
DRH: Dessie Referral Hospital
ETB: Ethiopian birr
MRN: Medical registration number
WHO: World Health Organization.
